# Three‐dimensional reconstruction of porous polymer films from FIB‐SEM nanotomography data using random forests

**DOI:** 10.1111/jmi.12950

**Published:** 2020-08-19

**Authors:** M. RÖDING, C. FAGER, A. OLSSON, C. VON CORSWANT, E. OLSSON, N. LORÉN

**Affiliations:** ^1^ RISE Research Institutes of Sweden, Biomaterials and Health Agriculture and Food Göteborg Sweden; ^2^ Department of Mathematical Sciences Chalmers University of Technology and University of Gothenburg Göteborg Sweden; ^3^ Department of Physics Chalmers University of Technology Göteborg Sweden; ^4^ AstraZeneca R&D Mölndal Sweden

**Keywords:** Controlled drug release, FIB‐SEM, Image analysis, Machine learning, Microstructure, Polymer films, Porous materials, Random forest, Segmentation

## Abstract

Combined focused ion beam and scanning electron microscope (FIB‐SEM) tomography is a well‐established technique for high resolution imaging and reconstruction of the microstructure of a wide range of materials. Segmentation of FIB‐SEM data is complicated due to a number of factors; the most prominent is that for porous materials, the scanning electron microscope image slices contain information not only from the planar cross‐section of the material but also from underlying, exposed subsurface pores. In this work, we develop a segmentation method for FIB‐SEM data from ethyl cellulose porous films made from ethyl cellulose and hydroxypropyl cellulose (EC/HPC) polymer blends. These materials are used for coating pharmaceutical oral dosage forms (tablets or pellets) to control drug release. We study three samples of ethyl cellulose and hydroxypropyl cellulose with different volume fractions where the hydroxypropyl cellulose phase has been leached out, resulting in a porous material. The data are segmented using scale‐space features and a random forest classifier. We demonstrate good agreement with manual segmentations. The method enables quantitative characterization and subsequent optimization of material structure for controlled release applications. Although the methodology is demonstrated on porous polymer films, it is applicable to other soft porous materials imaged by FIB‐SEM. We make the data and software used publicly available to facilitate further development of FIB‐SEM segmentation methods.

**Lay Description:**

For imaging of very fine structures in materials, the resolution limits of, e.g. X‐ray computed tomography quickly become a bottleneck. Scanning electron microscopy (SEM) provides a way out, but it is essentially a two‐dimensional imaging technique. One manner in which to extend it to three dimensions is to use a focused ion beam (FIB) combined with a scanning electron microscopy and acquire tomography data. In FIB‐SEM tomography, ions are used to perform serial sectioning and the electron beam is used to image the cross section surface. This is a well‐established method for a wide range of materials. However, image analysis of FIB‐SEM data is complicated for a variety of reasons, in particular for porous media. In this work, we analyse FIB‐SEM data from ethyl cellulose porous films made from ethyl cellulose and hydroxypropyl cellulose (EC/HPC) polymer blends. These films are used as coatings for controlled drug release. The aim is to perform image segmentation, i.e. to identify which parts of the image data constitute the pores and the solid, respectively. Manual segmentation, i.e. when a trained operator manually identifies areas constituting pores and solid, is too time‐consuming to do in full for our very large data sets. However, by performing manual segmentation on a set of small, random regions of the data, we can train a machine learning algorithm to perform automatic segmentation on the entire data sets. The method yields good agreement with the manual segmentations and yields porosities of the entire data sets in very good agreement with expected values. The method facilitates understanding and quantitative characterization of the geometrical structure of the materials, and ultimately understanding of how to tailor the drug release.

## Introduction

For 3D imaging of porous microstructures, combined focused ion beam and scanning electron microscope (FIB‐SEM) tomography (Inkson *et al*., 2001) is among the most powerful techniques. Using FIB‐SEM, substantially higher spatial resolutions can be obtained compared to, e.g. contemporary X‐ray computed tomography (X‐ray CT) systems. A 3D data set is acquired in a serial fashion where in‐between each image, a slice of the sample is milled away using the ion beam (the FIB) to reveal a new planar cross‐section. The imaging is performed using the electron beam of the scanning electron microscopy (SEM). This process is repeated up to the desired number of slices in the stack and size of the volume to be analysed.

FIB‐SEM tomography is used on a routine basis for highly conductive materials and ceramics, but for soft, poorly conducting and porous materials, the technique is less straightforward. The stack of images reveals the 3D microstructure of the sample, but there are particular challenges and artefacts of FIB‐SEM tomography that are qualitatively different from those of, e.g. X‐ray computed tomography. First, the most prominent challenge is the so‐called shine‐through effect when imaging porous materials; that the scanning electron microscopy images contain information not only from a planar cross‐section of the material but also from underlying, exposed subsurface pores. Consequently, FIB‐SEM produces a stack of 2.5D rather than 2D images. Second, charges due to the electron beam exposure can accumulate, observed as very bright regions in the images. This effect can be reduced by using a low electron beam energy and imaging using the backscattered electron signal (Holzer *et al*., 2004). Third, curtaining effects, i.e. lines parallel to the ion beam can be caused by local variations in hardness or thickness within the sample, resulting in varying ion milling rates locally and in effect a nonplanar cross‐section. This artefact can be reduced by lowering the milling rate or depositing a platinum coating on the sample surface (Giannuzzi & Stevie, 2005). Fourth, milled material still present in the chamber can be deposited back onto the cross‐section, known as redeposition, which can be reduced by a lower milling rate (Giannuzzi & Stevie, 2005). Fifth, shadowing effects occur if the surrounding material shadows the cross‐sections, i.e. blocks the electron beam path. This is addressed by milling trenches on both sides of the cross‐section prior to imaging (Holzer *et al*., 2004). Sixth, an artificial intensity gradient can occur in the images due to lower detection efficiency of the electron signal from the bottom of the slices, which in turn is due to the longer path of electrons from the beam to the detector (Joos *et al*., 2011). This can be compensated for at the experimental stage by changing the geometry of the sample (Schaffer & Wagner, 2008), but is more frequently compensated for by image processing (Taillon, 2016; Taillon *et al*., 2018). For a more comprehensive account of the experimental challenges of FIB‐SEM and a protocol for 3D imaging of soft, porous and poorly conducting materials, see Fager *et al*. (2020).

A plethora of different advanced image processing methods have been proposed to analyse and segment FIB‐SEM data, e.g. adaptive thresholding (Blayvas *et al*., 2006), level sets (Jørgensen *et al*., 2010), morphological image processing (Prill *et al*., 2013), local threshold backpropagation (Salzer *et al*., 2012, 2014), watershed segmentation (Taillon *et al*., 2018) and combinations of watershed, variance filtering and morphological operations (Reimers *et al*., 2019). Furthermore, a comparison of some approaches is performed in Salzer *et al*. (2015). Segmentation remains a challenging problem however, to a large extent because of the shine‐through effect that leads to two complications: uncertainty in the positioning of microstructural features along the axis perpendicular to the cross‐sections and an overlap of greyscale intensities between solid regions and pore regions.

In this work, we develop a segmentation method for FIB‐SEM data from ethyl cellulose porous films made from ethyl cellulose and hydroxypropyl cellulose (EC/HPC) polymer blends. The work is a continuation of the work in Fager *et al*. (2020), where the experimental parameters were optimized for milling and imaging of these soft, porous and poorly conductive materials. The ethyl cellulose and hydroxypropyl cellulose blend is used as a coating material on pharmaceutical oral dosage forms (tablets or pellets) to control drug release. During film formation, phase separation results in ethyl cellulose (EC)‐rich and hydroxypropyl cellulose (HPC)‐rich domains. HPC is water‐soluble, and hence leaches out when exposed to water, leaving a continuous network of pores that allows drug release. To design the coating for optimal controlled release, it is crucial to understand (i) how processing parameters influence microstructure and (ii) how microstructure influences transport of the drug through the coating. This understanding can be greatly increased by accurate quantification of the microstructure with respect to not only porosity but also geometry: pore size distribution, connectivity, tortuosity, etc. Here, we study three samples of ethyl cellulose and hydroxypropyl cellulose with varied HPC fraction, where the HPC phase has been leached out, resulting in a porous EC material. The image pixels are classified as solid (EC) or pore (leached out HPC) using a segmentation method based on scale‐space feature extraction combined with a random forest classifier. We show good agreement with manual segmentations performed by an expert. The methodology is demonstrated on these porous polymer films but the principle is applicable to FIB‐SEM data acquired from other soft porous materials as well. To facilitate further development of FIB‐SEM segmentation methods, we make all the data and Matlab (Mathworks, Natick, MA, U.S.A.) software used herein publicly available (Röding *et al*., 2020).

## Materials and methods

The phase‐separated polymer films consisting of ethyl cellulose (EC; Ethocel Standard premium, viscosity 10 cP, Dow Wolff Cellulosics GmbH, Bomlitz, Germany) and hydroxypropyl cellulose (HPC; Klucel Pharm HPC, viscosity grade LF, Ashland, Covington, U.S.A.) are produced as follows. Three 6% (w/w) solutions of both polymers combined are prepared, using 95% ethanol as solvent (Siepmann *et al*., 2007; Jansson *et al*., 2014; Fager & Olsson, 2018), sprayed onto a rotating drum, then removed and stored in a desiccator. The three samples have HPC weight fractions 22%, 30% and 45%, and are denoted HPC22, HPC30 and HPC45. Because the molecular weights of EC and HPC are very close, the volume fractions of HPC are nearly equal to the weight fractions. The HPC phase, which is water‐soluble, is leached out using stirred deionised water at ambient conditions for 24 h, yielding porous EC matrices. Finally, the resulting films are air dried. It is expected that the porosities of the final films will be close to 22%, 30% and 45%, provided that the HPC phase is fully leached out in all the samples (the lowest porosity sample, 22%, is close to the percolation threshold, Marucci *et al*., 2009, and might very well not be fully leached out).

FIB‐SEM tomography is performed using a TESCAN GAIA3 (TESCAN, Brno, Czech Republic), equipped with a gas injection system for platinum and carbon. The films are coated with palladium prior to insertion into the FIB‐SEM in order to reduce charging effects caused by the electron and ion beams. A platinum layer is deposited on the surface to reduce curtaining, and a U‐shaped trench around the cross‐section surface is milled to reduce shadowing, followed by cross‐section polishing. Serial sectioning is performed with a slice thickness of 50 nm and acquiring 200 slices for imaging of a 10‐μm‐thick section. Cross‐section imaging is performed using the mid‐angle back scattered electron (BSE) detector with an electron beam scan speed of 2 μs pixel${}^{-1}$ and a pixel size of 10 nm. We refer to Fager *et al*. (2020) for more experimental details.

## Results and discussion

We begin with preprocessing of the image data followed by manual segmentation of a small fraction of the data set. Then, we extract image features useful for segmentation. Using the manually segmented data, the segmentation is optimized with respect to hyperparameters of the classifier and data augmentation. Finally, the classifier is applied to the full data sets and postprocessing of the result is performed.

### Image preprocessing

Cross‐section images are aligned in ImageJ (Schneider *et al*., 2012) using the StackReg plugin and the Rigid Body method, which aligns the images based on translation and rotation only (i.e. no rescaling or general affine transforms). After alignment, equal sized volumes from all three samples are extracted, with resolution 2247 × 3372 pixels and 200 slices, comprising approximately 22.5 μm × 33.7 μm × 10.0 μm. The 16‐bit data are converted to [0,1] range.

**Fig 1:**
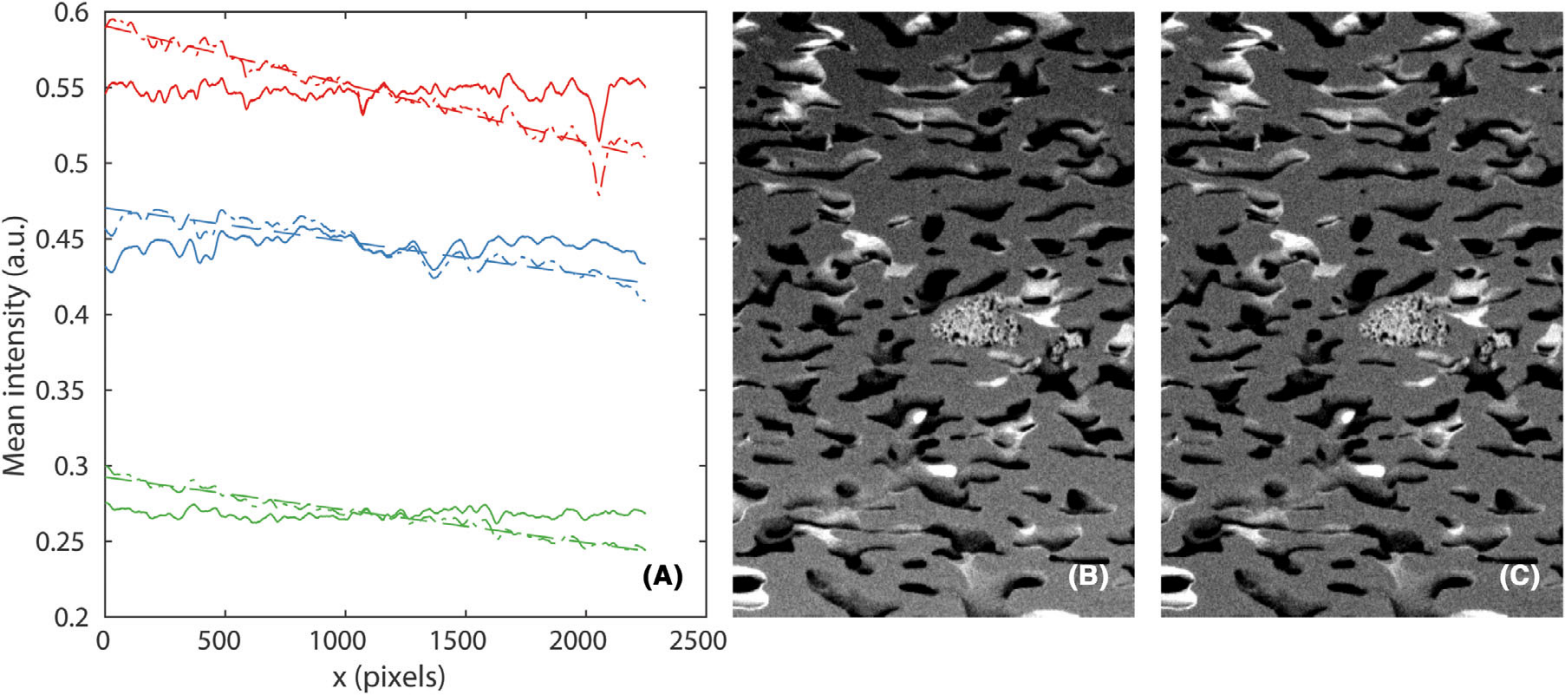
Intensity gradients present in the images are removed by fitting a linear function to the intensity profile using least squares, subtracting it and finally adding its mean value to retain approximately the original intensity range. In (A), the original intensity profiles (dash‐dot lines) are approximated by linear functions (dashed lines) which are subtracted to produce the corrected intensity profiles (solid lines), for HPC22 (red), HPC30 (green) and HPC45 (blue). In (B), a crop from an image of the original HPC30 data and in (C), a crop from the same image of the corrected HPC30 data are shown. The image intensities are rescaled to make the gradient clearly visible. The field of view in (B) and (C) is 22.5 μm × 15.0 μm.

Further, an intensity gradient is present in one direction (the *x* direction) of the images. It has been proposed earlier to 'normalize' the intensities of the slices orthogonal to the gradient direction by matching first and second moments of these (computed in the *y*–*z* plane), using the moments of the first *x* slice as reference values (Taillon, 2016; Taillon *et al*., 2018). Although this does remove the intensity gradient, it suppresses all first and second moment variations along the direction of the intensity gradient. Effectively, it hides part of the natural intensity variations that are induced simply by porosity fluctuations. These variations are not artefacts and should not be removed. Therefore, we propose an alternative solution. We exploit the fact that the intensity gradient is near‐linear and fit a linear function to the intensity profile using least squares, subtract it and finally add its mean value to retain approximately the original intensity range. Figure 1 shows some of the results of the correction; in (B), there is a clearly visible gradient, but subtracting the fitted linear function in (A), the gradient can be removed in (C).

It should be mentioned here that we did try the method of moment matching as well, concluding that it produces slightly worse results in terms of segmentation accuracy for our data.

### Manual segmentation

**Fig 2:**
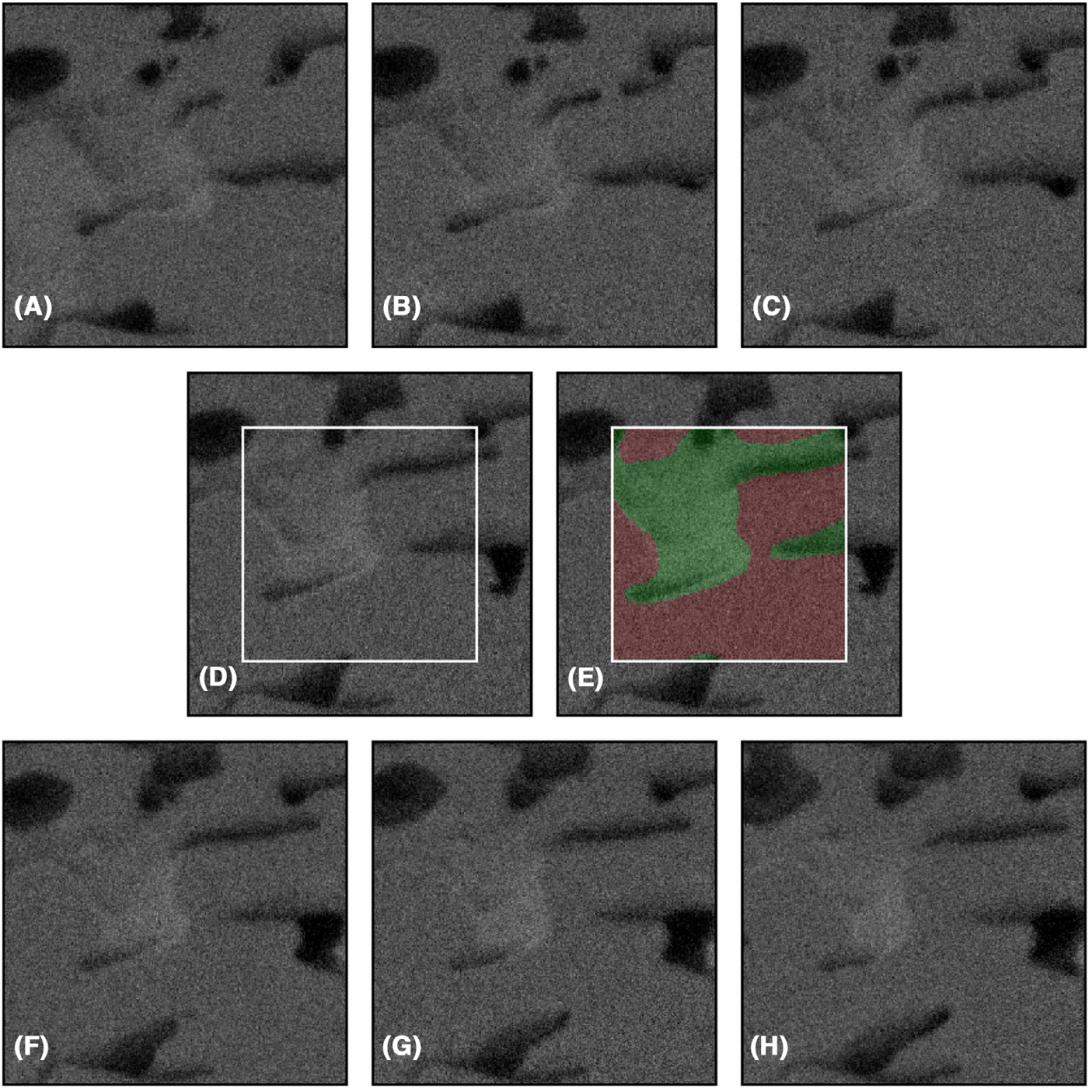
An example of a randomly selected square region of size 256 × 256 pixels from the HPC30 data set and the surrounding neighbourhood extracted for manual segmentation, showing (A)–(C) three slices before the slice of interest, (D) the slice to be manually segmented (inside the white square), (E) the resulting manual segmentation indicating solid (red) and pore (green) and (F)–(H) three slices after the slice of interest. Importantly, this figure also illustrates the so‐called shine‐through effect when imaging porous materials; that the image slices contain information not only from a planar cross‐section of the material but also from underlying, exposed sub‐surface pores. Indeed, solid material present in slices behind the slice of interest (originating from the slice in (A) or even further back) is visible in the slice of interest. As stated above in the text, focused ion beam and scanning electron microscope hence produces a stack of 2.5D images in the sense that there is a certain amount of depth information in each slice. The field of view is 3.84 μm × 3.84 μm.

For each data set, manual segmentation is performed by an expert. This is done only on a subset of the full data because manual segmentation of the complete data sets would take months. For each data set, 100 square regions of size 256 × 256 pixels are selected at random positions in random slices. This corresponds to ∼0.5% of the full data. Neighbourhoods around these of size 384 × 384 × 7 pixels, i.e. containing three adjacent slices in each direction are extracted (slices too close to any edges for this to be possible are avoided). The expert is provided with these neighbourhoods for manual segmentation, which is performed using MITK Workbench (Medical Imaging Interaction Toolkit, www.mitk.org) using careful manual tracing of the pore edges. The porosities as determined by manual segmentation for the three data sets are (showing mean and 95% confidence intervals; computed from the porosities of the 100 square regions, and assuming independence between those values) 21.72% ([20.28,23.17]), 29.54% ([27.70,31.38]) and 44.86% ([42.23,47.49])%. This is in close agreement with expected values. The values are somewhat lower than the expected values 22%, 30% and 45%, which should be obtained if the imaged volume is representative of the sample and if the HPC phase is fully leached out, but not significantly so. Figure 2 shows an example of such a neighbourhood and the resulting manual segmentation. In addition, it illustrates the shine‐through effect described above. To minimize the effect of fatigue and potential mistakes, the square regions are randomly shuffled in all subsequent steps of the image analysis.

### Feature extraction

**Fig 3:**
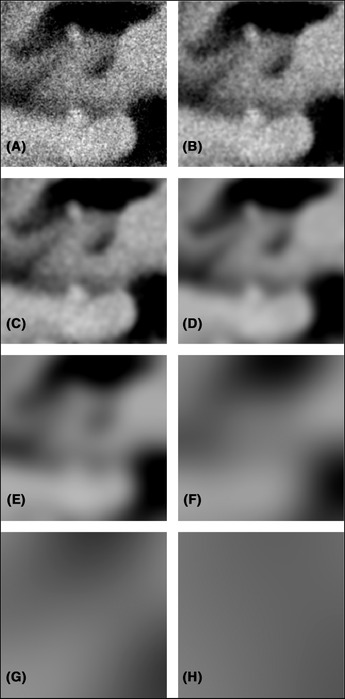
An example of the linear scale‐space feature space from the HPC45 data set, showing the same image with Gaussian smoothing applied for (A) σ=1, (B) σ=2, (C) σ=4, (D) σ=8, (E) σ=16, (F) σ=32, (G) σ=64 and (H) σ=128 pixels. The field of view is 2.56 μm × 2.56 μm.

We will segment the image data by classification of individual pixels as either solid, i.e. EC or pore i.e. (leached) HPC. On top of the raw image data, we use *x*–*y* neighbourhood information in each slice by extracting so‐called linear scale‐space features, i.e. a set of Gaussian smoothed images at different scales (Koenderink, 1984). We extract Gaussian filtered images using standard deviations σ = 1, 2, 4, 8, 16, 32, 64 and 128 pixels. The filter sizes are 4σ+1 except for σ=128 pixels, in which case the filter size is 2σ+1 to avoid too large edge effects near the data volume boundary. The rationale for the scale‐space features is that information about solid and pore phases can be found not only in the single pixel to be classified but in neighbourhoods of that pixel of varying sizes. Additionally, the same information is extracted from the adjacent five slices in each direction. Hence, information from 11 slices and 9 spatial scales (including the raw data, corresponding to σ = 0) is used. The total number of extracted features is therefore 99. Figure 3 shows an example of scale‐space feature images.

### Data split and classification algorithm

Of the manual segmentation data, of the 100 square regions for each data set, we use 60 for training, 20 for validation and 20 for testing. Hence, in total, 180 square regions are used for training, 60 for validation and 60 for testing. For each of the training, validation and test data sets, 2.5% of the available data (pixels) are extracted for machine learning, i.e. 294,912 samples for the training set and 98,304 samples for each of the validation and test sets (we refrain from using all available data in this step due to the computational workload, and because the data for adjacent pixels are strongly correlated). It is ensured through stratified random sampling that all three data sets have a 50/50 balance between the classes.

Classification is performed using random forests (Breiman, 2001). A random forest classifier constitutes a set of $n_{t}$ decision tree classifiers that collaborate through a majority voting rule (we let $n_{t}$ be odd to avoid voting ties). Each decision tree is constructed using Gini's diversity index (Breiman *et al*., 1984) for splitting. Further, each tree is trained on a bootstrap replica of the data, and uses $n_{f}$ randomly selected features. The depth and complexity of each tree is controlled by the minimum leaf size $n_{\mathrm{mls}}$. The raw output of the classifier is a score equal to the average vote. In our case, the classes are encoded as 0 (pore) and 1 (solid). The score can then be interpreted as a probability of belonging to the class solid. The score is thresholded (by default at 0.5) to yield a segmentation.

For assessing classification performance we use the (volume of the) intersection over (the volume of the) union, also known as the Jaccard index. Generally, it is defined by

(1) $$\mathrm{IoU}=\frac{\left|M\cap A\right|}{\left|M\cup A\right|},$$

where M and A are the manual and automatic segmentations. As is commonly done, we obtain a class‐symmetric measure by taking the average intersection over union: Let $M_{0}$, $M_{1}$, $A_{0}$ and $A_{1}$ be the manual and automatic segmentation of classes 0 (pore) and 1 (solid). The average intersection over union is then defined by

(2) $$\mathrm{mIoU}=\frac{1}{2}\left(\frac{|M_{0}\cap A_{0}|}{|M_{0}\cup A_{0}|}+\frac{|M_{1}\cap A_{1}|}{|M_{1}\cup A_{1}|}\right).$$

We also report accuracy, i.e. the proportion of correct classifications.

As a side note, we additionally consider Dice similarity coefficient,

(3) $$\mathrm{DSC}=2\frac{\left|M\cap A\right|}{\left|M|+|A\right|},$$

and binary cross‐entropy of the score

(4) $$\mathrm{BCE}=\langle y_{i}\log\left({\hat{y}}_{i}\right)+\left(1-y_{i}\right)\log\left(1-{\hat{y}}_{i}\right)\rangle ,$$

where $y_{i}$ is the true class (0 or 1) and ${\hat{y}}_{i}$ is the score (in the range $0\leq {\hat{y}}_{i}\leq 1$) for the ith pixel, and an average value is taken over all pixels. However, we find that these two similarity measures lead to the same models being selected in the hyperparameter optimization below, and hence stick to IoU and accuracy.

### Data augmentation

We investigate different training data augmentation and normalization schemes such as adding noise and blur, random anisotropic scaling, random linear intensity transforms and feature vector standardization. The only augmentation we find that provides an improvement in terms of validation set mIoU is the introduction of random linear intensity transforms in the following fashion. For each training sample, the local image neighbourhood is intensity transformed by

(5) $$I^{★}\left(x,y,z\right)=a+bI\left(x,y,z\right),$$

where a and b are random coefficients. We let both a and b be normal distributed, $a∼N(0,\sigma_{a})$ and $b∼N(1,\sigma_{b})$, and independent for each sample of the training set. Because the feature extraction is performed by linear filtering, these augmentation transforms can be applied to the already computed feature vectors in the training set. Therefore, the data augmentation can be included as part of the hyperparameter optimization below.

### Hyperparameter optimization

For the random forest classifier, we are interested in the following hyperparameters: The number of trees $n_{t}$, the number of features per tree $n_{f}$ and the minimum leaf size $n_{\mathrm{mls}}$. Because the resampling of the data set and selection of features for each tree is random, the optimization is also affected by random seed sensitivity. Initially, we include $n_{t}$ in the optimization and study the range $151\leq n_{t}\leq 301$ (only odd values in the range). However, because no significant improvement is found for increasing $n_{t}$ in that range, we fix $n_{t}=151$ to limit the computational workload. We perform a random search in the ranges $10\leq n_{f}\leq 40$ (after excluding the rest of the full range $1\leq n_{f}\leq 99$ beforehand) and $10\leq n_{\mathrm{mls}}\leq 50$. We also introduce the data augmentation and perform a random search over $\sigma_{a}$ and $\sigma_{b}$, where both are sampled from an exponential distribution with mean 0.01.

The hyperparameter optimization is run with and without data augmentation, investigating ∼1500 trained models in each case. The best model (with respect to validation set mIoU) with nonaugmented training data is found for $n_{t}=151$, $n_{f}=30$ and $n_{\mathrm{mls}}=28$. The best model with augmented training data is found for $n_{t}=151$, $n_{f}=28$, $n_{\mathrm{mls}}=14$, $\sigma_{a}=0.0009$ and $\sigma_{b}=0.0189$. The performance of the models is shown in Table 1. The model based on augmented training data leads to somewhat better generalization to the validation and test sets.It is worth pointing out that we find the same models to be the best also with respect to accuracy (as well as Dice similarity coefficient, and binary cross‐entropy for the score).

**Table 1:** Results for the best models (with respect to validation mIoU) that are found in hyperparameter optimization, without and with data augmentation. The model based on augmented training data leads to somewhat better generalization to the validation and test sets

	Train	Val	Test
*Without data augmentation*			
mIoU	0.9097	0.7719	0.7488
Accuracy	0.9527	0.8713	0.8564
*With data augmentation*			
mIoU	0.8619	0.7738	0.7636
Accuracy	0.9258	0.8726	0.8660

### Impact of the amount of training data

**Fig 4:**
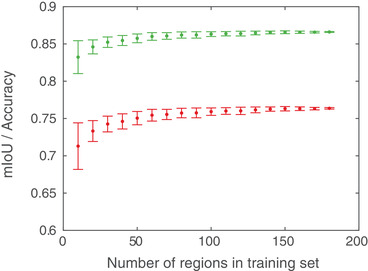
Test set mIoU (red) and accuracy (green) as a function of the number of square regions used for training. The results are based on 100 trained classifiers for each data set size (showing *m* ± SD).

One interesting aspect is the impact of the amount of training data on the performance. In an attempt to provide an answer, we train new classifiers with the optimal parameters identified above (with data augmentation) on random subsets of the 180 square regions used for training, and evaluate the test set mIoU and accuracy. More precisely, for random subsets of 10, 20, 30, ..., 180 square regions we train 100 new classifiers for each data set size [and, as before, 2.5% of the available data (pixels) are extracted for machine learning, for all training data set sizes]. The results will have random variation not only because of the randomness in the training data but also because of the inherent randomness of training a random forest classifier. Test results (*m* ± SD) as a function of training data set size are shown in Figure 4. As can be seen, both mIoU and accuracy have stabilized relatively well for substantially smaller amounts of training data than used herein. However, the amount of training data needed are highly dependent on the data set. In any case, the results indicate that the chosen training data set size is sufficient for the problem at hand.

### Analysis of the full data sets and postprocessing

We use the model based on augmented training data for analysis of the full data sets. For computation of features near the edges of the volume, the data is extended by mirroring. Prediction using the random forest model on a single slice (2247 × 3372 pixels) takes approximately 30 s (on a dual AMD Epyc 7542 setup with 64 cores and 256 GB memory), i.e. approximately 5 h in total. We evaluate the performance on the full square regions in the training, validation, and test sets (not only on the small random subsets extracted for machine learning). The results, for the individual data sets and combined, are shown in Table 2.

So far, we classify all pixels independently and use a score threshold of T=0.5. To spatially regularize the segmentation, we introduce a postprocessing step with Gaussian smoothing of the score array and thresholding. It is found that between‐slice smoothing yields no improvement, so we use within‐slice smoothing with standard deviation $\sigma_{xy}$ pixels and a threshold T. The smoothing and subsequent thresholding is followed by a second smoothing and finally a second thresholding, using the same parameters as in the first iteration, since this slightly improved the result. The parameters are tuned using derivative‐free optimization with respect to validation mIoU, yielding the optimal values $\sigma_{xy}=2.796$ pixels and T=0.4495. The rationale behind having T≠0.5 is to compensate for the fact that the data sets extracted for machine learning are class‐balanced, whereas the full data sets are clearly not (because of the porosity being <50%, it is expected that the optimum will be at T<0.5). As a final postprocessing step, we remove small connected objects (smaller than 100 pixels) both from the pore phase and the solid phase; this has a negligible impact on the overall performance but produces smoother and less noisy binary structures. The final results, for the individual data sets and combined, are shown in Table 3. We see that both mIoU and accuracy for all data sets increase after postprocessing, and that all estimated porosities decrease.

**Table 2:** Results before postprocessing for the individual data sets as well as combined. The results are based on the full square regions in the training, validation, and test sets, not only on the small random subsets extracted for machine learning

	Train	Val	Test
*HPC22*			
mIoU	0.7750	0.7464	0.7473
Accuracy	0.9068	0.8919	0.8985
Porosity (%), manual	22.07	22.00	20.42
Porosity (%), automatic	24.15	24.77	22.23
*HPC30*			
mIoU	0.8578	0.8280	0.8013
Accuracy	0.9339	0.9195	0.9045
Porosity (%), manual	29.76	29.14	29.27
Porosity (%), automatic	32.11	31.26	32.43
*HPC45*			
mIoU	0.8255	0.7108	0.6924
Accuracy	0.9051	0.8310	0.8202
Porosity (%), manual	44.17	49.62	42.20
Porosity (%), automatic	47.05	49.24	46.62
*Combined*			
mIoU	0.8272	0.7689	0.7520
Accuracy	0.9153	0.8808	0.8744

**Table 3:** Results after postprocessing for the individual data sets as well as combined. The results are based on the full square regions in the training, validation and test sets, not only on the small random subsets extracted for machine learning

	Train	Val	Test
*HPC22*			
mIoU	0.7790	0.7539	0.7522
Accuracy	0.9123	0.8999	0.9050
Porosity (%), manual	22.07	22.00	20.42
Porosity (%), automatic	21.35	21.87	19.59
*HPC30*			
mIoU	0.8650	0.8324	0.8080
Accuracy	0.9384	0.9231	0.9097
Porosity (%), manual	29.76	29.14	29.27
Porosity (%), automatic	30.41	29.48	30.39
*HPC45*			
mIoU	0.8330	0.7186	0.7089
Accuracy	0.9100	0.8365	0.8329
Porosity (%), manual	44.17	49.62	42.20
Porosity (%), automatic	44.58	46.04	42.80
*Combined*			
mIoU	0.8339	0.7752	0.7616
Accuracy	0.9202	0.8865	0.8825

**Fig 5:**
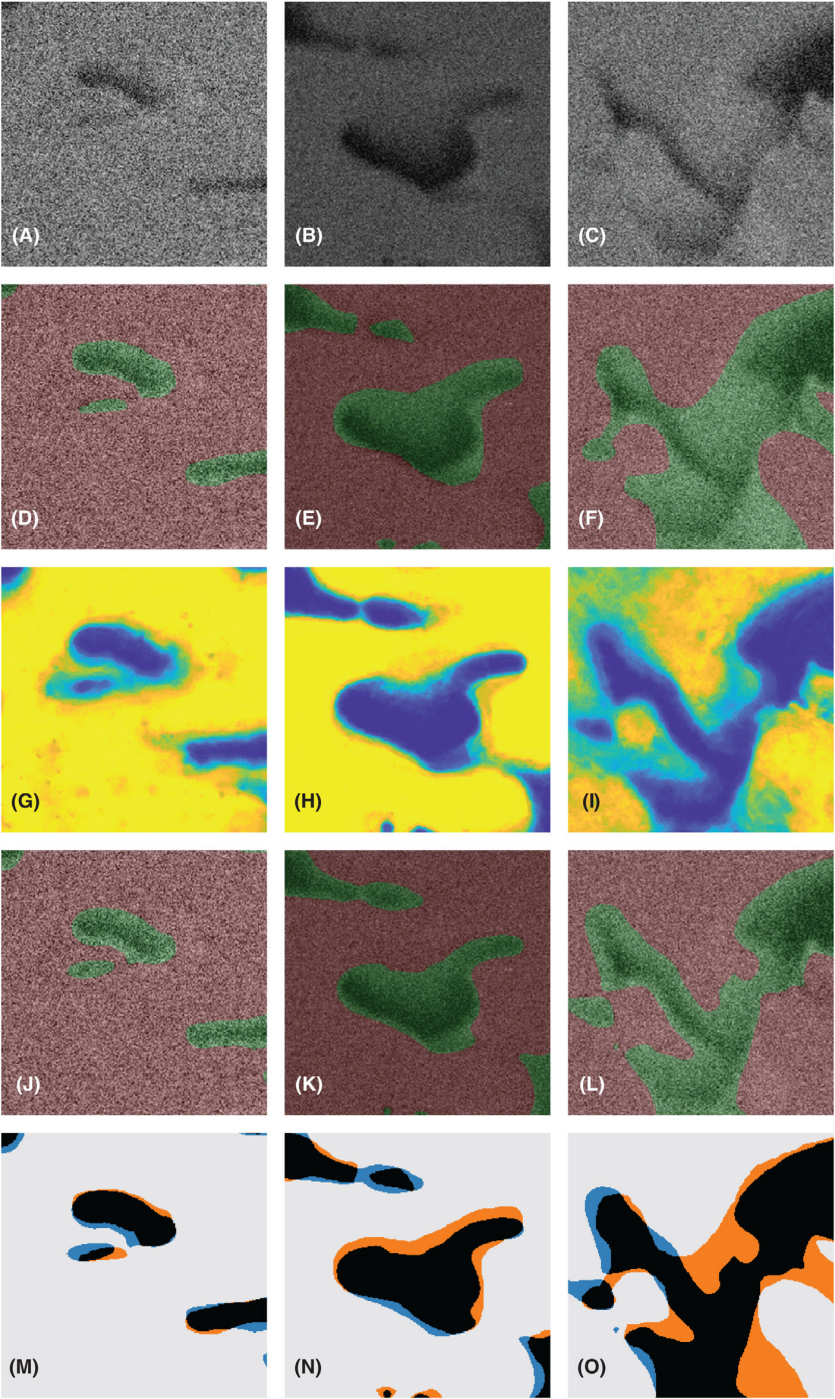
Comparison of manual and automatic segmentation for one region each of the test data from the HPC22 (left column), HPC30 (centre column) and HPC45 (right column) data sets. In (A)–(C), the image data are shown. In (D)–(F), the manual segmentation is superimposed on top of the image data, showing pores (green) and solid (red). In (G)–(I), the raw score is shown. The raw score can be interpreted as the probability of a pixel to belong to the solid phase (blue ≈ 0, green ≈ 0.5, yellow ≈ 1). In (J)–(L), the automatic segmentation is superimposed on top of the image data, showing pores (green) and solid (red). In (M)–(O), an overlay of the manual and automatic segmentations is shown, with correctly classified pores (black), correctly classified solid (off‐white), pores incorrectly classified as solid (orange) and solid incorrectly classified as pores (blue). The field of view is 2.56 μm × 2.56 μm.

**Fig 6:**
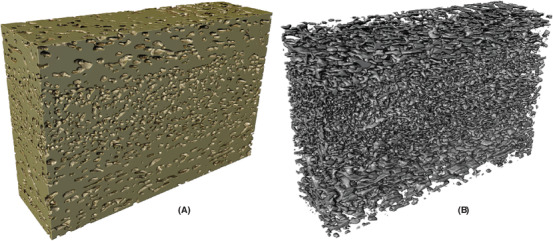
Automatically segmented structures for HPC22, showing (A) the solid phase (ethyl cellulose) and (B) the pore phase (leached hydroxypropyl cellulose).

**Fig 7:**
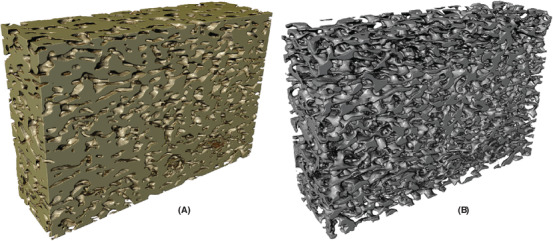
(Automatically segmented structures for HPC30, showing (A) the solid phase (ethyl cellulose) and (B) the pore phase (leached hydroxypropyl cellulose).

**Fig 8:**
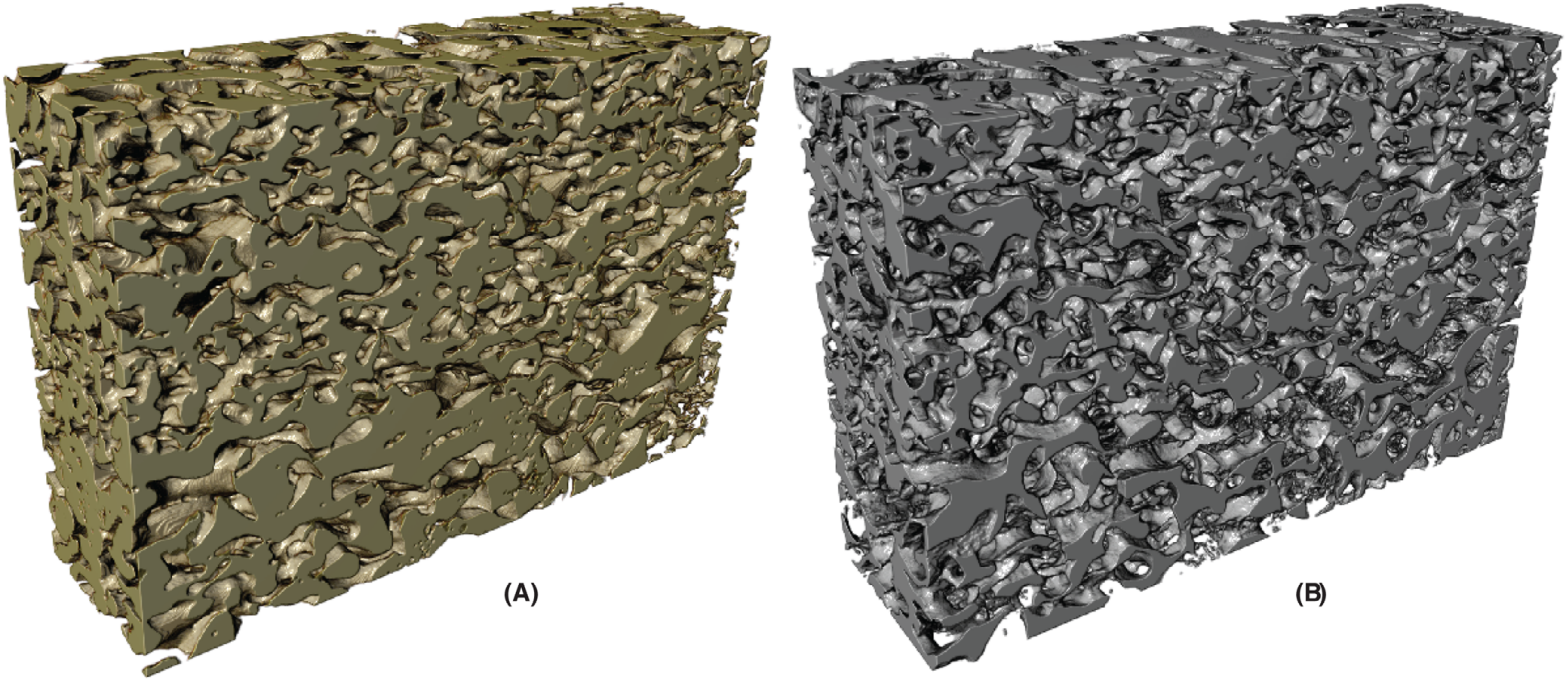
Automatically segmented structures for HPC45, showing (A) the solid phase (ethyl cellulose) and (B) the pore phase (leached hydroxypropyl cellulose).

In Figure 5, one example of the segmentation result from each of the data sets are shown, with a comparison of the manual and automatic segmentation. The final automatic segmentations for the whole data volumes are shown in Figures 6, 7 and 8, where the identified solid structures and pore structures are visualized separately. We reiterate that the porosities as determined by manual segmentation for the three data sets are (showing mean and 95% confidence intervals) 21.72% ([20.28,23.17]), 29.54% ([27.70,31.38]) and 44.86% ([42.23,47.49]). These values are computed from the porosities of the 100 square regions, merging training, validation and test data, and assuming independence between the 100 values. The corresponding porosities as estimated from automatic segmentation are 21.10% ([19.89,22.32]), 30.22% ([28.32,32.12]) and 44.52% ([42.01,47.02]). According to these results, the manual and automatic segmentations are not significantly different in terms of porosity. However, we emphasize that the manually segmented regions constitute less than 0.5% of the total data and might not be representative in terms of porosity for the whole data.

For the entire data sets, the porosities as estimated from the automatic segmentations are 20.17%, 32.27% and 44.36%. Assuming that the true porosities are exactly 22%, 30% and 45%, the estimated porosities deviate slightly, which could be attributed to incomplete leaching and/or difficulties in segmentation due to the shine‐through effect. The HPC22 sample is close to the percolation threshold (Marucci *et al*., 2009) and hence, some HPC‐rich domains might not be connected to the rest of the HPC phase and be trapped. However, the obtained porosities are in good agreement with the expected porosities. Also, given that the samples are very heterogeneous, we cannot ascertain that the true porosities of the imaged volumes of our samples are exactly 22%, 30% and 45%.

As a comparison to the proposed approach, we evaluate the performance of a global thresholding. Optimizing the combined mIoU of the training and validation sets for each data set individually (since there are no hyperparameters we might as well combine the training and validation sets in this case) with respect to the threshold value, we obtain test mIoU values for the three data sets of 0.4768, 0.6162 and 0.4962. Further, the estimated porosities of the test sets are considerably worse (14.59%, 23.12% and 35.42%) than for the proposed approach. This supports our decision to use machine learning methods for segmentation of these data.

## Conclusion

We have developed a segmentation method for FIB‐SEM data samples of EC porous films produced from EC/HPC polymer blends where the HPC phase has been leached out to obtain a porous network structure, and quantified the microstructures of three different samples with different porosities. The image pixels have been classified as solid or pore using a random forest classifier, resulting in good agreement with manual segmentations performed on parts of the data sets by an expert. Further, the automatically segmented structures have porosities that are in good agreement with the expected porosities of the structures. The method enables quantitative characterization and facilitates subsequent optimization of the material structure for controlled release applications. The methodology is not specific to the data from porous polymer films studied here. Indeed, the principles are applicable to other soft porous materials imaged by FIB‐SEM, provided that new manual segmentation data are available and that training of the classification algorithm is performed specifically for the new data. We make the data and software used publicly available to facilitate further development of FIB‐SEM segmentation methods, of benefit for the materials studied herein as well as for others. Interesting further work would be to incorporate also the unlabeled data in the classifier training in a semisupervised fashion to better exploit the amount of data acquired, and to implement other advanced classifiers such as convolutional neural networks for performance comparison.
